# Researching on the Effect of Input Parameters on the Quality and Manufacturability of 3D-Printed Cellular Samples from Nylon 12 CF in Synergy with Testing Their Behavior in Bending

**DOI:** 10.3390/polym16101429

**Published:** 2024-05-17

**Authors:** Martin Koroľ, Jozef Török, Peter Pavol Monka, Petr Baron, Beata Mrugalska, Katarina Monkova

**Affiliations:** 1 Faculty of Manufacturing Technologies with a Seat in Presov, Technical University in Kosice, 080 01 Presov, Slovakia; jozef.torok@tuke.sk (J.T.); peter.pavol.monka@tuke.sk (P.P.M.); petr.baron@tuke.sk (P.B.); 2 Faculty of Technology, Tomas Bata University in Zlin, Nam. T.G. Masaryka 275, 760 01 Zlin, Czech Republic; 3 Faculty of Engineering Management, Poznan University of Technology, J. Rychlewskiego 2, 60-965 Poznań, Poland; beata.mrugalska@put.poznan.pl

**Keywords:** Nylon 12 CF, cellular structures, additive technology, input parameters, bending

## Abstract

The study of cellular structures and their properties represents big potential for their future applications in real practice. The article aims to study the effect of input parameters on the quality and manufacturability of cellular samples 3D-printed from Nylon 12 CF in synergy with testing their bending behavior. Three types of structures (Schwarz Diamond, Shoen Gyroid, and Schwarz Primitive) were selected for investigation that were made via the fused deposition modeling technique. As part of the research focused on the settings of input parameters in terms of the quality and manufacturability of the samples, input parameters such as volume fraction, temperature of the working space, filament feeding method and positioning of the sample on the printing pad were specified for the combination of the used material and 3D printer. During the experimental investigation of the bending properties of the samples, a three-point bending test was performed. The dependences of force on deflection were mathematically described and the amount of absorbed energy and ductility were evaluated. The results show that among the investigated structures, the Schwarz Diamond structure appears to be the most suitable for bending stress applications.

## 1. Introduction

The development and application of cellular structures have spread across many industries in recent years. This is because of the extraordinary properties they have. The production of such structures with regularly distributing cells and deterministically specified geometry was not possible using conventional manufacturing methods such as turning, milling, drilling and the like. This was the main reason for the low use of cellular structures in industrial components as a whole in the past. It was only after the introduction of additive technologies that it became possible to produce complex shapes such as cellular structures. In the case of plastics, currently, the most used production methods within additive technologies include the fused deposition modeling (hereinafter FDM) technique, and in the case of metals they include direct metal laser sintering (DMLS) [1,2].

Since the final quality of the part is influenced by many parameters, a very important part of the process of producing bodies with a porous structure of the required quality is the preparatory phase, which consists of several stages. These parameters can include not only the topological characteristics of the structure (basic cell geometry, basic cell distribution and volume fraction) but also the material characteristics and technological conditions of 3D printing (layer thickness, nozzle diameter, printing speed, substrate, workspace temperatures, 3D printing strategy, etc.). All of this subsequently affects the properties of the manufactured components and their service life when they operate in technical practice. The quality of the selected components consequently affects not only their mechanical properties, and thus their operability, but also the safety of the entire device into which the components are to be implemented [3,4,5]. Therefore, the investigation of the combination of parameters used in the production process is an important aspect of research when studying their mechanical properties.

## 2. State of the Art

Cellular structures can be found in nature all around us. Due to their properties such as their ability to reduce weight while maintaining strength or absorbing energy and transferring heat, their use is ranges across several engineering fields (automotive, mechanical engineering, aeronautics, medicine, etc.). A cellular structure can be considered a medium that consists of a solid and a gas phase. The latter consists of an interconnected network of rigid struts or plates that form the edge0s and surfaces of the cells [6,7,8,9].

**Figure 1:**
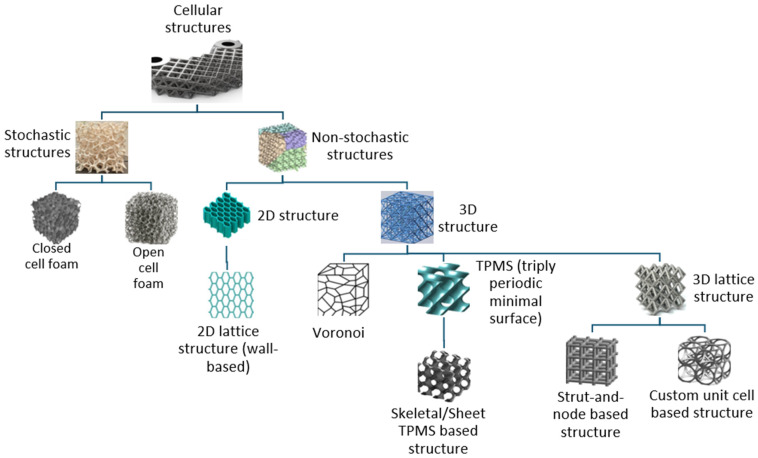
Categories of cellular structures.

The classification of cellular structures can be divided into several subgroups. Initially, in additive manufacturing, this classification concerns the selection of the appropriate unit cell. The basic cell is selected based on what the model or proposed test sample is used for. As such, cellular structures are divided into two main categories: stochastic and non-stochastic structures. The detailed distinctions can be seen in Figure 1 [10].

Many researchers in the world have worked on the issue in the past, but there are also a number of research teams working on the issue today. In the past, even before researchers tested porous structures, they mainly tested honeycomb structures, which was also addressed, for example, by Tohid Ghanbari-Ghazijahani. The research pointed out the differences between two materials, namely wood and polylactic acid (hereafter PLA), which at the time were considered materials with high potential for use in several industries. In the research, several beams made of these materials were used with different volumes and distributions inside the investigated specimens. These were subsequently examined and tested until they were fully deformed or broken. Ultimately, these results proved that plastics such as PLA have a higher strength compared with wooden webs and results were obtained that were promising for the further pursuit of the subject in the future [11]. 

Another who has worked on the subject is Flaviana Calignano, who describes the actual mechanical properties of parts made from Nylon filaments that are reinforced with carbon fiber. In the results of that research, differences in values were seen that did not match the material data sheets from different material suppliers. The hardness and strength of the specimen printed from Nylon material with a 20% carbon fibere content differed due to several factors such as the printing direction, and percentage of filler, while the flexibility was mainly affected by the printing direction [12].

Vuong Nguyen-Van’s research team looked at the reinforcement of concrete beams in 2021 in their paper “Performance of concrete beam reinforced with 3D printed Bioinspired primitive scaffold subjected to three-point bending”. Since most concrete beams are reinforced with steel bars, his team decided to replace the steel bars with a concrete TPMS structure and test its flexural strength. The structure they decided to use was Primitive. In their research, three concrete beams of the same size (including unreinforced and reinforced with single- and double-layer thin-walled forms, respectively) were tested. These were made specifically for the three-point bending test only and to compare them with each other. The results of the research showed significantly improved flexural properties. When the beam was reinforced with TPMS of the Primitive structure, its flexural strength improved by 35% for the single-layer reinforcement and 125% for the double-layer reinforcement. Moreover, the research also indicated that concrete beams reinforced with a TPMS structure exhibit smooth softening in bending and show increased ductility [13]. 

The research on energy absorption and the mechanical properties exhibited by specimens made from TPMS structures are elaborated upon in the article by Shaun Ormiston. Specifically, this study dealt with the fabrication of composite lattice structures reinforced with a second (secondary) material. The lattice structures were modeled into cubic, cylindrical, rod, and dog-bone geometries while the volume fraction inside the specimen was varied by 25%, 50% and 75%, respectively. The structures were extruded from Nylon, which contains carbon fibers, and reinforced with glass fiber. The results showed, in this case, that the glass-fiber-reinforced structures with a reduction in the amount of material to a total of 25% had similar or better properties compared with structures with a 75% volume of the material without the addition of glass fiber reinforcement [14]. 

The research by Ibrahim M. Alarifi investigated the effects of changing the raster orientation (0°, 45° and 90°) using a 3D printer. Two materials were used in the research, namely Carbon Fiber Nylon (hereafter referred to as Nylon CF) and Glass Fiber Nylon (hereafter referred to as Nylon GF), the samples of which were then subjected to a three-point bending test. As the research showed in the initial results, the highest stiffness was found for the Nylon GF samples, which were tested at room temperature, whereas on the other hand, the Nylon CF samples were found to have higher elasticity and lower flexural strength than the former [15].

Cui et al. studied the effect of 3D printing process parameters and their optimization for improving the mechanical properties of a fabricated cellular structure made of fiber-reinforced PLA composite material and Kevlar filament. They investigated the effect of process parameters in 3D printing and tested the fabricated models in three-point bending. The three-point bending tests showed the relationship between process parameters and mechanical properties. They showed higher flexural strength when using Kevlar filament [16].

Myers et al. dealt with the optimization of process parameters in the fabrication of Schoen Gyroid and Schwarz Primitive cellular structures from PLA material. Analyzing the geometrical deviations of the fabricated physical models with respect to the nominal ones, they pointed out the significant influence of flow rate and layer height on the accuracy of the models. They also pointed out the significant effect of layer height and printing speed on the fabrication time [17].

Tao et al. studied the optimization of 3D models of cellular structures made of PLA composite material with wood and flour admixtures. The study highlighted the differences between the nominal model used for FEA analysis and the actual fabricated physical model. They pointed out the formation of voids within the printed model and their significant effect on the mechanical properties, and modified the 3D model to correspond to the actual compression test results against the FEA analysis [18]. 

The improvement in the mechanical properties of TPMS porous structures was investigated by Zhang et al. They proposed the use of adaptive thickness to reduce anisotropy [19]. Zhu et al. designed, fabricated and mechanically tested TPMS cellular structures to optimize their mechanical properties. They showed an improvement in the mechanical properties of the thickness-optimized samples [20]. Yu et al. studied the fabrication and mechanical property testing of TPMS cellular structures fabricated using additive technologies [21].

Abueidda et al. examined the mechanical properties of Gyroid cellular structures. They used FEA analysis and the Arruda–Boyce finite deformation elasto-viscoplastic model, which they compared with real testing conditions. The FEA analysis corresponded with the experimental results and indicated suitable mechanical properties compared with those of Neovius and Primitive [22].

As technologies develop very quickly, while at the same time new materials have appeared, the possibility to incorporate cellular structures not only in industrial components but also in devices of daily use is becoming an increasingly likely reality. It is therefore very important for designers to know their properties and behavior under different types of loads. The presented research aims to determine the bending properties of samples with three selected types of cellular structures (Schwarz Dimond, Schoen Gyroid and Schwarz Primitive) made of Nylon 12 CF, pointing out that the input parameters can affect not only the quality of the product but also its mechanical and other properties. Since there are many 3D printers and materials available on the market today that can be combined across a range of applications, the article offers insight into several problems that have to be solved in the 3D printing process before the actual production of final samples is achieved for experimental bending tests.

Based on the state of the art, researchers have recognized the value of porous structures that can be implemented in many applications. However, to the best of the authors’ knowledge, none of the available studies has yet investigated the behavior of selected cellular structures made of carbon fiber-reinforced Nylon under bending loads while solving problems in setting the input parameters for the available 3D printer. This represents an opportunity for contributions to the field and novel research with the potential for subsequent possibilities to implement this know-how and experience in simulations of the production process, as well as into numerical analyses of the behavior of lightweight components.

## 3. Materials and Methods

### 3.1. Characteristics of the Selected TPMS Structures

For a basic investigation of the influence of process parameters on the fabrication of Nylon Carbon Fiber cellular structures and the specification of their bending properties, Schwarz Diamond, Schwarz Primitive (also called Schwarz P) and Schoen Gyroid structures, which belong to the group of so-called triply periodic minimal surfaces (TPMSs), were chosen in the present research. These are surfaces in three-dimensional space that minimize the surface area for a given bounding curve. At the same time, they are triple-periodic, i.e., they repeat in three independent directions, and this periodicity gives them crystallographic symmetry. They are characterized, for example, by having a zero-mean curvature at each point. This means that they are locally flat and have no sharp edges [23,24,25].

The selection of the type of samples was related to extensive research on the behavior of cellular structures that has been ongoing at the authors’ workplace for several years. The authors have dealt with the production of cellular samples produced from both metal materials (the samples were made of aluminum alloy AlSi10Mg or Inconel 718 using DMLS technology) and plastics (the samples were made of ABS and PLA using FFF technology). Concerning cell topology, the results showed that the way cells are distributed in the test body (radial or orthogonal) does not have as significant an effect on its mechanical properties as the type of structure and wall thickness (or cross-section of the strut) when maintaining the same volume fraction. Furthermore, the research showed that sharp transitions between individual struts in lattice structures (mutual position at a certain angle) act as notches and reduce their mechanical properties, in contrast to structures of triply periodic minimal surfaces (TPMSs), in which the transition between the walls in their spatial architecture is continuous and smooth. The results of already performed experimental tests on tension and compression, comparing the properties of the Schwarz Diamond, Schoen Gyroid and Schwarz Primitive structures, highlighted the dominance of the Schwarz Diamond structure, so in the presented research, the authors tried to determine the behavior of the structures under bending loads and verify whether the Schwarz Diamond structure would again be the best in terms of bending. This method of stressing is very common in practice, whether in the civil engineering industry for the load-bearing constructions of buildings or for the static stressing of shafts in mechanical engineering, but it can also be found in various frame constructions (e.g., conveyors and gantry cranes), such as in the aviation industry.

**Figure 2:**
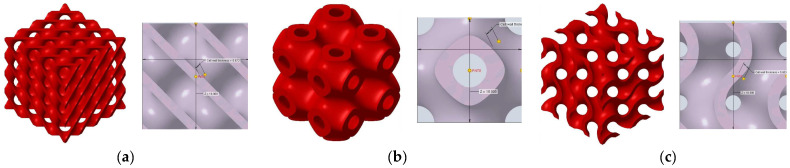
Investigated structures’ arrangement with their basic cell*s*: (**a**) Schwarz Diamond; (**b**) Schwarz Primitive; (**c**) Schoen Gyroid.

Figure 2 shows the structures chosen for this research along with the geometries of their basic cell. The Schwarz Diamond structure (Figure 2a) has simple two-dimensional sixfold symmetry, which means that each vertex of this surface is connected to six vertices in its surroundings, and this structured is formed by a lattice with simple symmetric surfaces [26,27,28].

In contrast to the Schwarz Diamond structure, the Schwarz Primitive (Figure 2b) structure differs in that each vertex of the surface is connected to five vertices in its surroundings. Geometrically, it is a structure that is formed by a grid where each face is symmetrical to the others [29,30].

The Schoen Gyroid (Figure 2c) is a structure that is characterized by triple-period and minimum-area surfaces. The structure offers high planar distributivity and an open post-surface morphology that can be exploited for efficient interaction with other substances or the environment [31].

### 3.2. Material of the Samples 

The specimens for flexural testing were made from Nylon 12 Carbon Fiber, (MakerBot, New York, NY, USA), hereafter referred to as Nylon 12 CF, which is created by blending Nylon 12 and short carbon fibers. It has high strength, resistance to wear, chemicals and moisture. The carbon fibers in this case give the material extra strength and stiffness. Thanks to its durability and strength, it is used in several sectors such as aviation (aircraft components such as wings, fuselage and landing gear), automotive (parts such as bumpers, fenders and bodywork), mechanical engineering (bearings, gears or various types of housing) and sports (skis, snowboards and tennis racquets). Nylon 12 CF material is characterized by high abrasive properties, and therefore a hardened steel or ruby nozzle is recommended for 3D printing. The advantages of the material include its high strength, durability and its lightness, making it resistant to chemicals. The disadvantages are its lower ductility and temperature resistance [32,33].

### 3.3. Production of Samples

Virtual 3D models of selected TPMS structures were created using PTC Creo Parametric 10 software, and they were exported into an STL file for further processing. The MarkerBot Method X printer (MakerBot, New York, NY, USA), available in the authors’ workplace, was employed with MarkerBot Cloud software 3.10.1 for the specimens’ fabrication. 

**Figure 3:**
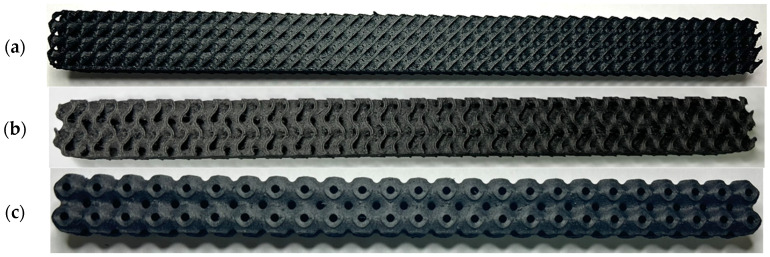
Samples for experimental testing: (**a**) Schwarz Diamond, (**b**) Schoen Gyroid and (**c**) Schwarz Primitive.

For the investigation of the bending properties, the samples (shown in Figure 3), measuring 20 × 20 × 250 mm with selected TPMS structures, were produced with sizes of a basic cell of 10 × 10 × 10 mm and a volume fraction of *V_f_* = 30%, which was specified based on Equation (1) [34]:

(1) $$V_{f}=\frac{V_{R}\;}{V_{C}}100\;(\%)$$

where *V_R_* is the material volume used for structure production, and *V_C_* is a total volume of the sample. 

The fabrication of specimens for testing the flexural loading properties of the structures was preceded by extensive research into the input parameters (described in more detail in Section 3), which had a significant influence on the final quality of the experimental specimens. For these purposes, samples measuring 20 × 20 × 20 mm were produced while maintaining the same cell size of 10 × 10 × 10 mm, which was intended for the final samples for bending.

### 3.4. Experimental Testing of Specimens under Bending Loads

**Figure 4:**
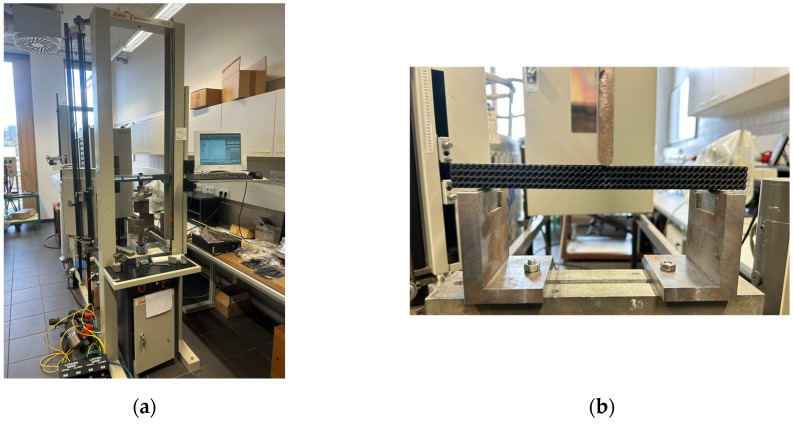
Test set-up; (**a**) Zwick 1456 machine; (**b**) a specimen positioning within bending test.

The flexural loading behavior of the porous specimens was tested in accordance with the ISO 178:2019 standard [35] on a Zwick 1456 (Ulm, Germany) testing machine (Figure 4a) at a temperature of 20 °C and with humidity at 50%. The supporting span was *l* = 200 mm, and the pressure thumb had a radius of 10 mm. The specimen set up is shown in Figure 4b.

Based on the measured data, the Young´s modulus, amount of absorbed energy and the ductility indices were evaluated.

## 4. Effect of Input Parameters on the Quality and Producibility of the Specimens

To implement structures into components that work in real practice, it is first of all necessary that the structures be produced repeatedly with good quality, and then it is necessary to know their properties and behavior in real conditions. Only by setting and observing the correct parameters and conditions will it be possible to ensure the reliability and safety of the operation of such a lightweight component. Testing low-quality samples with a lot of defects and without the required characteristics of shape and accuracy would therefore be meaningless. In the presented research, the authors tried to point out, in a simplified and shortened way, the importance of all basic aspects affecting the implementation of shape-complex cellular structures into lightweight components, which include the properties of the basic material, the conditions of sample production and the determination of load resistance characteristics.

During the first attempts to produce experimental samples to test their bending behavior, several problems were encountered concerning their manufacturability and their low quality. It was therefore necessary to carry out extensive preliminary research, the goal of which was to find the causes of sample production failures and to determine the influence of input parameters on their quality.

Possible reasons for failure were identified: insufficient quality and properties of the input material—filament (mechanical properties and humidity); the basic characteristics of the sample topology (the minimum volume fraction and need for supports during 3D printing); the influence of 3D printing conditions (the temperature of the working environment, method of material feeding, heat treatment process and positioning of samples in the workspace of the 3D printer). The influence of all mentioned parameters was gradually investigated, and, if necessary, the primary set values/methods were adjusted to ensure that the final samples were of sufficient quality for bending testing.

To save material, reduced specimens, measuring 20 × 20 × 20 mm were produced for this purpose, but with the same cell size of 10 × 10 × 10 mm, as was intended for the final experimental specimens stressed under bending.

### 4.1. Verification of Filament Properties

In the first step of identifying the causes of the poor quality of the samples and the failure of their production (when production could not be completed), the properties of the material Nylon 12 CF (which was delivered to the authors’ workplace in the form of a filament with a diameter of ϕ1.75 mm in the state of winding on spools and vacuum-packed with silicate inside) were verified, with the manufacturer declaring that it is a dried material. The properties of Nylon 12 CF stated in the material sheet provided by the producer are listed in Table 1. 

Nylon 12 CF is characterized by high susceptibility to moisture; therefore, it was necessary to check that the moisture content of the material does not reach a value that would invalidate results after measurement or that would impair the quality of the printed product. Moist filament can cause this when exposed to the temperatures used in 3D printing; moisture in the filament will expand and eventually volatilize. This greatly disrupts the normal extrusion pattern of 3D printing and will likely result in quality problems in the final print. As a result, the layers are printed incompletely, with defects ranging from small spots in the layers to layers that have entire gaps or holes in them [38,39]. One indication that this is due to moisture in the filament is that the effect persists throughout the printing process, creating an uneven surface, which occurred in the case of the research presented.

**Figure 5:**
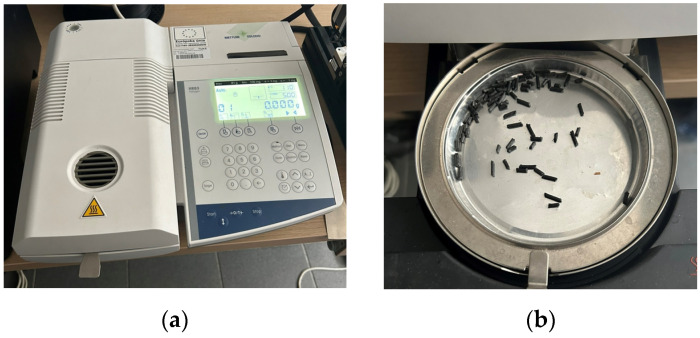
Filament moisture measurement: (**a**) Halogen HR83 moisture measuring instrument; (**b**) the material that was tested in the weighing pan.

The moisture content of the material was investigated using Halogen HR83 (Mettler Toledo, Columbus, OH, USA), shown in Figure 5, which works on the principle of thermogravimetry. 

The material was chopped into small pieces and placed in a weighing pan, which was then placed in the drying chamber of the analyzer. The balance accurately measured the weight of the sample before drying, and then the material was dried using a halogen heater included in the instrument. During drying, the sample was weighed every 30 s. Once the sample weight was stable, the analyzer calculated the moisture content, resulting in a value for the moisture content of the sample representing a percentage of the wet weight and indicated with a negative sign [40].

**Figure 6:**
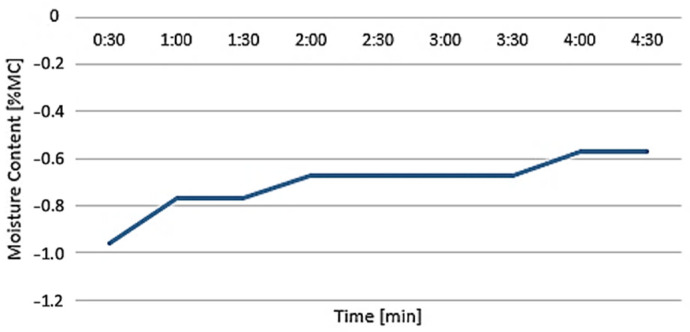
Measurement of moisture content of material Nylon 12 CF.

The temperature and measurement times were set to the prescribed values for drying Nylon 12 CF material, namely 110 °C and 5 min, respectively. The weight of the material that was placed in the weighing pan was 1.044 g, and after drying, the weight of the material was 1.038 g. The difference in this case was minimal (0.006 g), and the final moisture content value was 0.57%MC (Figure 6), where %MC is the amount of moisture present in a material, represented as a percentage of the material’s mass, calculated in accordance with Equation (2) [41]:

(2) $$MC=-\frac{WW-DW}{WW}100(\%)$$

where MC is moisture content, WW is wet weight and DW is dry weight. 

Considering the measurement results, it was possible to conclude that the material met the requirements for 3D printing; therefore, its moisture content should not be the cause of failure in the production process or of poor quality of the samples.

**Figure 7:**
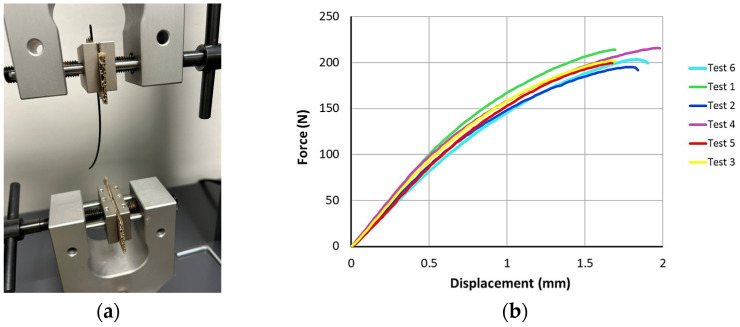
Tensile testing of filament: (**a**) tensile test set up; (**b**) measured force-displacement dependences.

Subsequently, the mechanical properties of the supplied filament were verified. The filament properties (ultimate strength, yield strength, Young’s modulus and ductility) under tensile loading were tested on Testometric X350-5 (Rochdale, UK) (Figure 7a) in accordance with ASTM D638 [42] at an ambient temperature of 19 °C and with a jaw speed of 50 mm/min, where the filament length was 100 mm, and testing was repeated 6 times.

The measured force dependences on displacement are presented in Figure 7b. The average ultimate tensile strength reach a value of 85.35 MPa, which corresponds to the value provided by the manufacturer in the material data sheet. 

### 4.2. Investigation of the Manufacturability of Nylon 12 CF Cellular Slurries

#### 4.2.1. Determination of the Volume Fraction of the Material

Once the filament properties were verified, the next phase of the research focused on the manufacturability of the samples with the selected cellular structures. Since one of the challenges of the implementation of porous structures is to lighten the components (while maintaining other required criteria related to physical and functional properties), it was also an effort in the presented research to keep the volume fraction of the material as low as possible. For the determination of the minimum volume fraction, *V_f_*, and for the investigation of the manufacturability of cellular structures made of Nylon 12 CF, volume fractions in the range of 10% to 40% were chosen, while the specimens were manufactured with dimensions of 20 × 20 × 20 mm. The change in volume fraction was controlled by varying the wall thickness in the structure while maintaining the cell size at 10 × 10 × 10 mm.

The wall thicknesses for each type of structure and for the given material volume fractions were defined using virtual 3D models in the PTC Creo 10.0 software environment and are listed in Table 2.

The fabricated structures were observed under a Celestron microscope with a resolution of 10×–150×, and photographs of the observations along with a description of the defects are shown in Table 3.

The setting value of the volume fraction, *V_f_* = 30%, for the final samples with cellular structures intended for bending testing was determined gradually, while the volume fraction of 10% was considered the minimum for the production of TPMS structures from plastic based on the experience of the authors gained from previous research. However, the given material is specific (due to carbon fiber reinforcement), and after the first attempts to make samples with this volume fraction, it was observed that their quality was very poor. Therefore, the volume fraction was gradually increased and the quality of the produced samples was checked.

As can be seen from Table 3, the deficiencies are mainly visible at a glance for the models containing a lower volume fraction (10% and 20%), for each of the selected TPMS structures. The most common error that occurred was associated with omitted layers or layers that did not interlock. Another of the visible defects was the edges of the sample characterized by a large amount of unevenness, especially at a 10% volume fraction for all three selected TPMS structures.

With a subsequent increase in material volume to 30%, the surface quality and detail improved significantly, and defects and irregularities were largely eliminated. Where imperfections were present, these were most often at the edges of the specimens where the carbon fibers did not adhere sufficiently to the basic structural unit of the specimen. 

It could be concluded that the samples at *V_f_* = 30% showed good quality, but it was worth investigating the quality at a 40% volume fraction and critically assessing whether or not the potential improvements in quality would balance out the material’s consumption and weight increase for future applications. It turned out that the difference in quality between samples with 30% and 40% volume fractions was only minimal, even for the Schwarz Diamond structure (Table 3); the paradox was that at a 40% volume, gaps were visible on the surface that were not observed at a 30% volume fraction. 

Despite the occasional occurrence of minor defects, it was possible to conclude that the quality of the samples with *V_f_* = 30% was comparable to the quality of samples with *V_f_* = 40%, while the differences were only minimal. Considering the above, a volume fraction of 30% was chosen for the production of final samples with cellular structures, which were intended for the study of bending properties.

#### 4.2.2. Impact of Support Structures on Manufacturability

TPMS-type structures are self-supporting, so no support material should be required for 3D printing. On the other hand, as mentioned in the previous chapter, due to the reinforcing fibers in the matrix of the base material, the geometry of the structures and the surface of the samples appeared not “clean” enough, and therefore the question arose as to whether or not support structures, especially used in additive manufacturing processes for samples with complex geometries, could help to improve the quality of the investigated samples generated with the help of cellular structures. Therefore, within the framework of the presented research, samples were also fabricated with the SR-30 Support Filament material, and its removal after fabrication was re-incorporated via dissolution at 65 °C using MakerBot Method Wash Tank (MakerBot, New York, NY, USA). The temperature was set based on the Nylon 12 CF material manufacturer’s recommendations so that only the support material, which was from MakerBot METHOD X SR-30 Support Filament, was dissolved.

Table 4 shows examples of samples with a *V_f_* = 30% volume fraction produced with and without the support material.

By examining the samples and comparing them, it was possible to conclude that the samples produced with the support material did not show a significant increase in quality compared with the samples produced without the support material. 

In view of the above, as well as due to the higher economic and time requirements for producing specimens with support material (higher material consumption, time consumed the for preparation and removal of structures, etc.), the final specimens for flexural testing were produced without support structures and additional support material.

### 4.3. Effect of Production Conditions on the Quality of Samples

#### 4.3.1. Effect of Ambient Temperature on Sample Quality

In the context of the research steps already carried out, and considering the findings of deficiencies related to the lack of material in the individual layers and the associated probable lack of adhesion of the material, research into the effect of the temperature of the printer’s working environment on the quality of the samples was subsequently carried out in the next phase.

Insufficient adhesion of the applied layers results in a heterogeneity of their properties and in the behavior of the samples under load, which was also proven by certain authors in their research [39]. In the case of plastic materials, this means that there was insufficient heating of the previous layer and mixing of the material in the new layer with the material in the previous one, which is currently being resolved by the authors with patents PP50069-2022 and PCT/SK2023/050030, and utility model PUV50104-2022, titled “Method of heat treatment of materials with control of spatial arrangement” [43,44,45]. Due to physical principles, it was therefore not important to decrease the temperature; on the contrary, it was necessary to increase the basic temperature recommended by the manufacturer and then compare the achieved quality of the samples.

The base temperature of the 3D printer chamber during the production of the Nylon 12 CF samples was chosen to be 60 °C, which is the temperature recommended by the material manufacturer and listed in the material sheet. For comparison of the print quality results, temperatures of 65 °C and 70 °C were further selected.

The comparison of the quality of the samples was carried out through observation under Celestrom 10×–150× Digital Microscope, with the samples printed at 65 °C showing an improvement in the surface quality and continuity of the layers (Table 5). A further temperature increase of 5 °C brought about a further deterioration in quality, with more defects on the surface and edges, and in layer continuity.

#### 4.3.2. Effect of Filament Feeding Method on Sample Quality

Another problem that had to be addressed during the sample production process was the frequent jamming of the material. As it was a specific material not only characterized by high abrasiveness, but also lower elasticity and high brittleness compared with materials such as ABS, PLA or PETG, while the base matrix of the material contained carbon particles, problems arose with the unwinding of the filament and its smooth feeding. This problem did not arise when other materials were used in the printer, so effects such as nozzle fouling or material build-up on the walls of the tube in the extruder could be eliminated.

**Figure 8:**
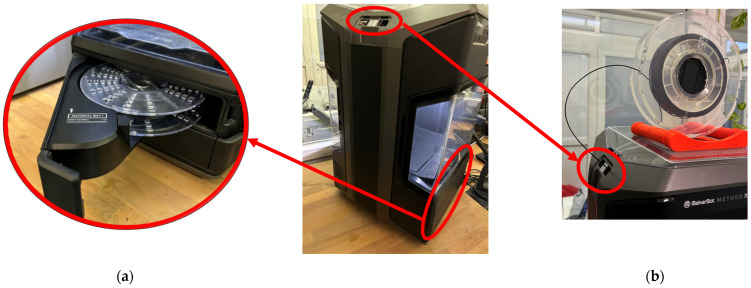
Filament feeding method: (**a**) placement of the material in the tray at the bottom of the printer*;* (**b**) placement of the material in the rack at the top of the printer.

By analyzing the problem and observing the behavior of the filament during the 3D printing of the samples, it was found that the cause of filament jamming was its deposition. This type of printer offers two ways to deposit the filament spool. Since Nylon 12 CF is highly susceptible to moisture, the primary choice was to place the material in the tray at the bottom of the printer (Figure 8a), which was closed. However, the properties of the filament, combined with the long path of the filament into the nozzle, caused problems with unwinding and with the smoothness of the filament dispensing into the extruder. Using a second method of placing the material in a rack at the top of the printer solved the problem (Figure 8b).

Other settings of the production parameters were based on the recommendations of both the filament manufacturer and the printer, and after verifying their suitability for the production of the selected cellular samples, the resulting print parameters were set to the values shown in Table 6. 

**Figure 9:**
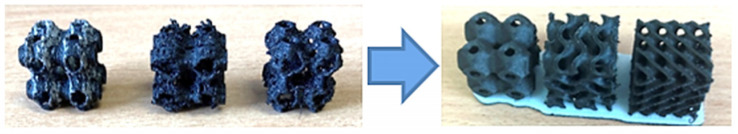
Results of improved technological conditions (Nylon 12 Carbon Fiber).

The result of the complex process of setting the input parameters was a significant improvement in the quality of the samples compared with that of the initially produced ones, as shown in Figure 9.

**Figure 10:**
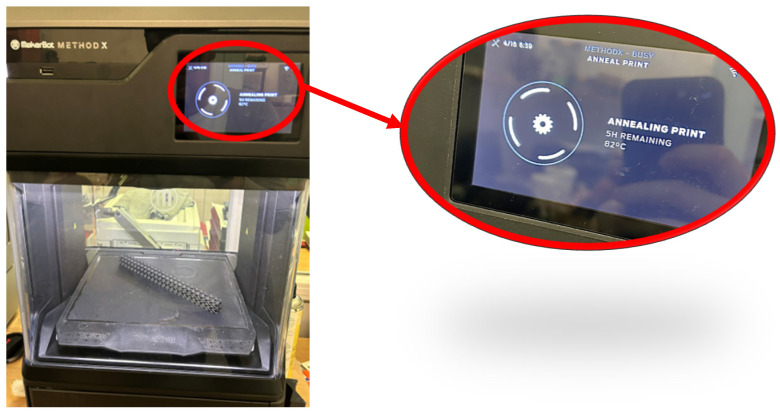
Annealing process of a sample inside of the workspace of the 3D printer machine.

Following the UltiMaker (Utrecht, The Netherlands) filament manufacturer’s recommendation, the fabricated samples were subjected to a low-temperature annealing process to remove stresses as part of the post-processing process, which was carried out at 82 °C for 5 h (Figure 10) [46].

#### 4.3.3. Effect of Sample Placement in the Printer Workspace

**Figure 11:**
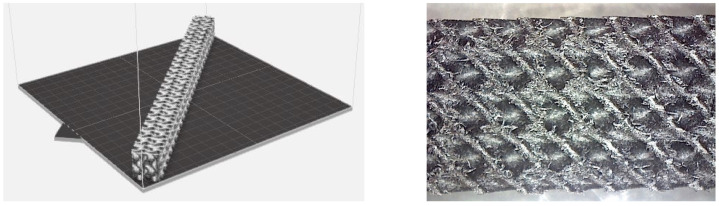

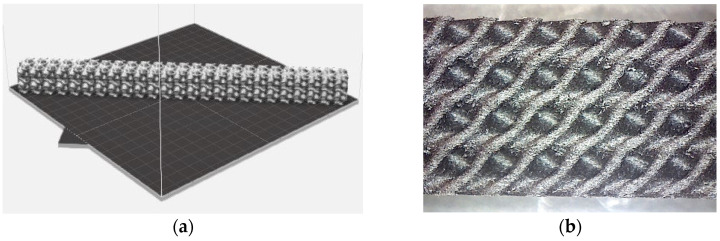
(**a**) Saving the sample in software as part of preprocessing before printing; (**b**) surface of the sample after printing under a Celestrom microscope at 40× magnification and with a 2 MP CMOS resolution.

After tuning the input conditions, it was necessary to produce the final specimens for flexural testing measuring 20 × 20 × 250 mm. Due to the 152 × 190 × 196 mm workspace of the 3D printer, the models were positioned diagonally on the work pad, and both directions were tested. As can be seen in Figure 11, the results clearly indicated that the positioning of the sample in the workspace had an impact on its quality. Since we used a closed printer, the influence of external conditions was excluded. A possible cause of sample surface deterioration, which will need to be considered when producing other types of samples, is the airflow generated inside the printer, which may cause defects associated with the faster cooling of the individual layers or filament at the point of deposition.

## 5. Testing of Specimens under Bending Load

### 5.1. Experimental Results 

For flexural testing, nine specimens in total with a 30% volume fraction were produced (three specimens each for all three structure types studied—Schwarz Diamond, Schoen Gyroid and Schwarz Primitive). 

**Figure 12:**
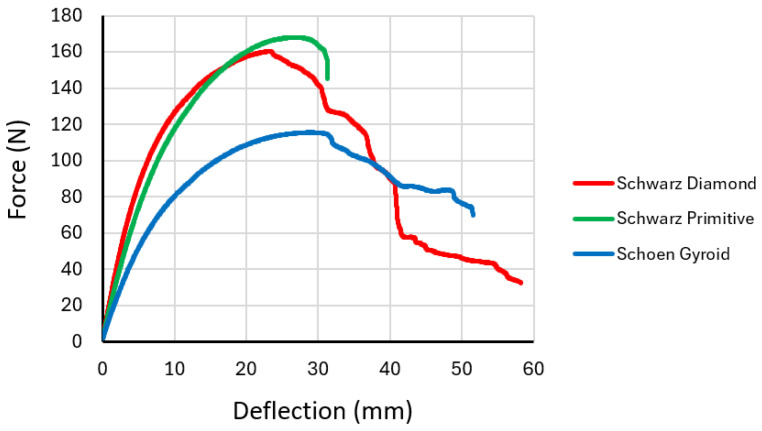
Representative force versus deflection curves obtained from experimental tests of specimens with cellular structures under bending loading.

During testing, the force versus deflection dependencies were recorded, the representative curves of which are plotted in Figure 12, with the results of the repeated testing of the same type of specimens showing comparable values and the dependency curves differing only minimally. 

The measured force dependences on deflection were for individual structures described by a polynomial function with a high coefficient of determination (R^2^) as follows:

Schwarz Diamond (R^2^ = 0.9952)

*y* = −0.0000004*x*^6^ + 0.0000626*x*^5^ − 0.0041344*x*^4^ + 0.1314913*x*^3^ − 2.3513441*x*^2^ + 26.5747373*x* − 1.1968865 (3)

Schoen Gyroid (R^2^ = 0.9949)

*y* = 0.0025*x*^3^ − 0.3042*x*^2^ + 10.413*x* + 4.3398 (4)

Schwarz Primitive (R^2^ = 0.9999)

*y* = −0.0000004*x*^6^ + 0.0000287*x*^5^ − 0.0011107*x*^4^ + 0.0345008*x*^3^ − 0.9437999*x*^2^ + 18.5008764*x* + 1.0870565 (5)

The Schwarz Primitive structure required the highest force to break the specimen at 168 N, and the Schoen Gyroid structure required the lowest (115.6 N), but in terms of deflection, the highest value of 58.25 mm was measured for the Schwarz Diamond structure. The results indicates that the Schwarz Diamond structure is the most ductile structure and that the Schwarz Primitive structure exhibits the most brittle behavior amongst the structures investigated from the material.

In terms of linear elasticity, the entire deformation work is converted into elastic tension energy. Based on the energy approach, the three-point flexural test can also be used to determine the modulus of elasticity, *E* (Young’s modulus, MPa), the bending stiffness (N/m) and the flexural rigidity (Nm^2^). If the elastic deflection, *u_e_*, is expressed by the equation

(6) $$u_{e}=\frac{F_{e}l^{3}}{48EI}(\mathrm{mm}),$$

then Young’s modulus can be expressed as

(7) $$E=\frac{F_{e}l^{3}}{48u_{e}I}(\mathrm{MPa}),$$

where *F_e_* denotes the applied elastic force (N), *l* is a supporting span and *I* is the area moment of inertia.

**Figure 13:**
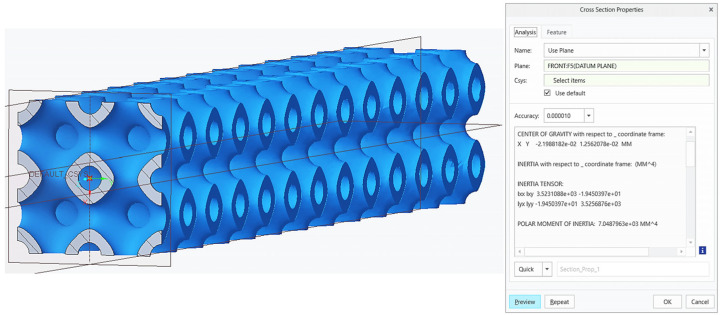
Specification of the area moment of inertia, *I*.

The moduli of elasticity in flexure for individual types of cellular structured beams were calculated in accordance with Equation (7), and they are listed in Table 7. For the calculations, the area moment of inertia of the specimen, *I*, was determined using the software PTC Creo, as shown in Figure 13, while the elastic force, *F_e_*, and elastic deflection, *u_e_*, were specified based on measured data. 

As can be seen from Table 7, the highest values of evaluated parameters that characterize the strength and plasticity of the structures were achieved for the Schwarz Diamond structure, and the lowest values were obtained for the Shoen Gyroid structure.

### 5.2. Energy Absorption and Ductility Assesment

The behavior of the selected cellular structures during testing was closely assessed using the amount of energy absorption and ductility indexes [47]. 

**Figure 14:**
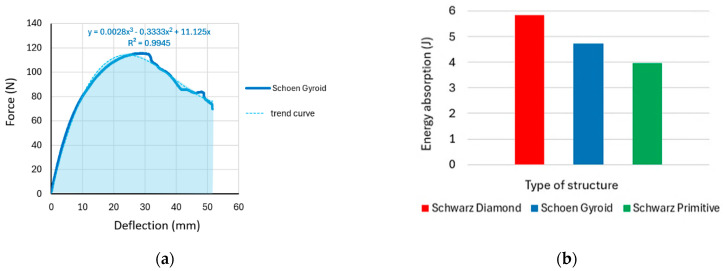
(**a**) Energy absorption during bending; (**b**) energy absorption during bending, elastic; ductility indexes, *µ_d_*, based on the deflection value at the proportionality limit. *µ_E_* is expressed as the quotient of the total and elastic energy.

The total energy absorption was calculated as the area under the force–deflection curve [48] by integrating the trend equation, which was expressed by a polynomial function, as seen in Figure 14a. The results for all three types of samples with cellular structures are presented in the histogram in Figure 14b.

The ductility of the beams was evaluated via two indices: *µ_d_* and *µ_E_*. The index *µ_d_* represents the ratio between the deflection at the ultimate load, *u_u_* (mm), and the deflection at the elastic limit, *u_e_* (mm), according to Formula (8) [49]:

(8) $$\mu_{d}=\frac{u_{u}\;}{u_{e}}$$

The ductility index, *µ_E_*, represents the quotient of the total and elastic energy, and it is expressed by Equation (9) [50,51]:

(9) $$\mu_{E}=\frac{1}{2}\left(\frac{W_{tot}}{W_{e}}+1\right)$$

- *W_tot_* is the total energy absorbed by the sample during bending (J);
- *W_e_* is the elastic energy (fraction of total) absorbed by the sample up to the elastic limit (J).

**Figure 15:**
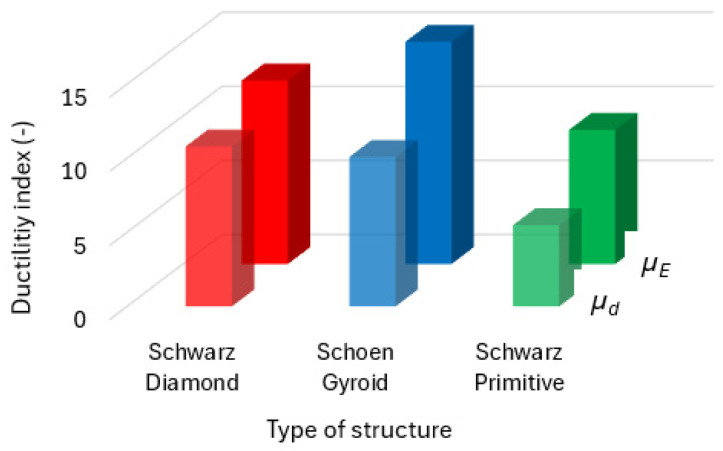
Ductility indexes, *µ_d_*, based on the deflection value at the proportionality limit. *µ_E_* is expressed as the quotient of the total and elastic energy.

Results for both calculated types of indexes were plotted, and they are presented in Figure 15.

It can be seen from the histograms in Figure 14b and Figure 15 that the Schwarz Diamond structure can absorb the greatest amount of energy under stress, while the Schwarz Primitive is able to absorb the smallest amount. Ductility indexes pointed out similar results, although for the quotient of the total and elastic energy, the Shoen Gyroid structure achieved the maximal value. The results indicate that the most brittle behavior among the studied structures was shown by the Schwarz Primitive and that the most ductile behavior was shown by the Schwarz Diamond structure.

## 6. Conclusions

Porous structures can bring many advantages to a component. In addition to being lightweight, they can save the amount of material used and provide components with extraordinary properties in terms of maintaining functionality, reliability and safety. It is therefore very important to know their behavior in advance even before they are introduced into real practice.

The aim of this paper was to investigate three types of porous structures that belong to the so-called group of triply periodic minimal surfaces and to compare their bending properties. This study was carried out in synergy with a comprehensive preliminary investigation of the effect of the input parameters of 3D printing (the FDM technique) on the quality of samples produced from Nylon 12 CF material using a selected 3D printer available in the authors’ workplace.

To set up the input parameters, the samples were produced using the FDM technique with sizes of 20 × 20 × 20 mm within the preliminary investigation, while for the study of bending properties, the sizes of cellular specimens were 20 × 20 × 250 mm. 

The results showed that for a given combination of materials and a 3D printing machine, the minimum volume fraction of the selected cell samples was *V_f_* = 30% while maintaining good sample quality. At this volume fraction, supports do not play a sufficient role in improving quality, so the samples were made to be self-supporting.

It was also found that setting the temperature to 65 °C in the 3D printer workspace during sample production resulted in better sample quality compared with that under the 60 °C temperature recommended by the filament manufacturer. Two other issues that had an impact on the quality of the structures were investigated within the presented research, i.e., the type of storage of the spool with a filament during the operation of the printer and also the positioning of the sample on the pad.

By experimentally testing the bending behavior of the selected cellular structures, it was found that although the highest force (168 N) needed to cause failure in sample was measured for the Schwarz Primitive structure, this structure showed the most brittle behavior. On the contrary, the largest amount of energy (5.84 J) was able to be absorbed by the sample of the Schwarz Diamond structure, which from a comprehensive point of view appears to be the most suitable for applications with Nylon 12 CF under bending stress.

The obtained results and experience will be able to be used in the near future in the simulation of the 3D printing process and the behavior of structures using numerical analyses.

## Figures and Tables

**Table 1:** Properties of Nylon 12 CF [36,37].

Properties	
Density	1.5 g/cm^3^
Melting point	180–190 °C
Temperature resistance (continuous)	−40 °C až 120 °C
Temperature resistance (short term)	180 °C
Tensile modulus of elasticity	10 GPa
Tensile strength	83.5 MPa
Elongation at break	10%
Impact toughness	50 kJ/m^2^

**Table 2:** Wall thicknesses for individual structure types and material volume fractions.

Volume Fraction	Type of Structure	Cell-Wall Thickness
10%	Shoen Gyroid	0.52 mm
	Schwarz Diamond	0.36 mm
	Schwarz Primitive	0.43 mm
20%	Shoen Gyroid	0.70 mm
	Schwarz Diamond	0.55 mm
	Schwarz Primitive	0.88 mm
30%	Shoen Gyroid	1.02 mm
	Schwarz Diamond	0.83 mm
	Schwarz Primitive	1.33 mm
40%	Shoen Gyroid	1.37 mm
	Schwarz Diamond	1.12 mm
	Schwarz Primitive	1.79 mm

**Table 3:** View of samples under a Celestrom microscope (Torrance, CA, USA) with a 40× magnification and 2 MP CMOS resolution.

Structure	Volume Fraction	View under the Microscope	Detail of Errors	Error Description
Diamond	10%	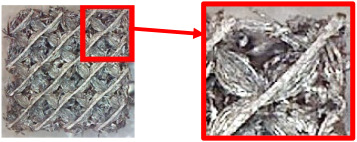		Excessively large gaps between layers
	20%	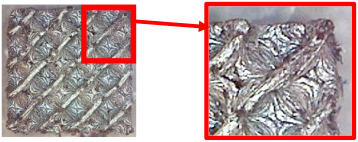		Tasseled surface on the edges of the sample
	30%	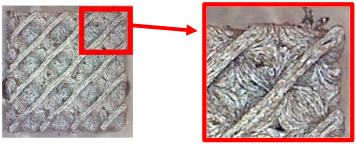		Better continuity of layers, slightly fringed edges
	40%	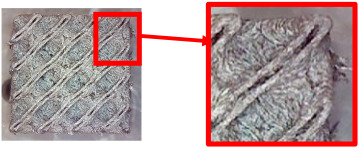		Large gaps between layers
Gyroid	10%	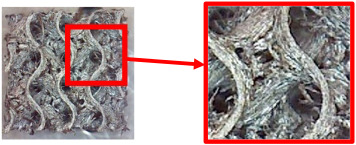		Excessively large omission of material caused by thin walls
	20%	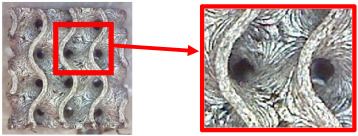		Slightly better surface of the sample compared to 10% + frayed ends of the sample
	30%	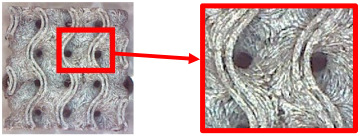		Gaps between layers on the surface
	40%	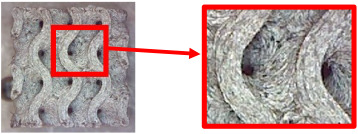		Better layer continuity versus lower volume fraction
Primitive	10%	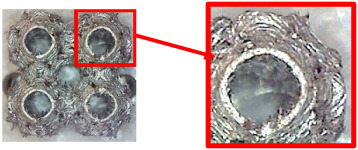		Very frequent omission of details and layering of material has too-large gaps
	20%	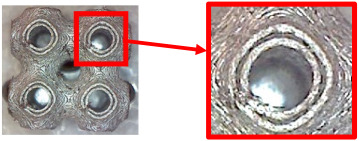		Excessively large gaps between layers
	30%	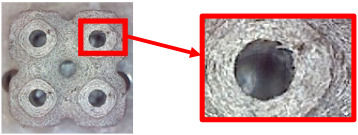		Visibly better surface, better printed details, and minimal surface defects
	40%	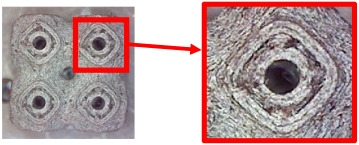		Visible omission of material between layers and reduced compactness

**Table 4:** Comparison of surface quality after printing with/without support material using a Celestrom microscope with a 40× magnification and 2 MP CMOS resolution.

Type of Structure	Sample Made with Support Material	Sample Made without Support Material
Diamond 30%	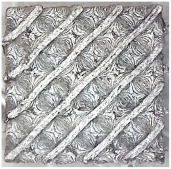	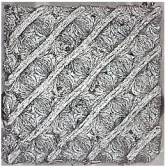
Gyroid 30%	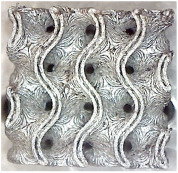	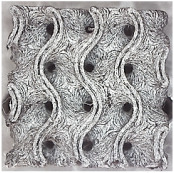
Primitive 30%	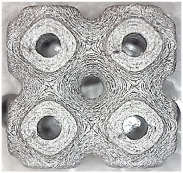	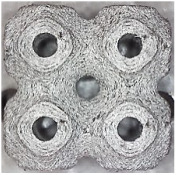

**Table 5:** View of samples under Celestrom microscope at different chamber temperatures, at a 40× magnification and with a 2 MP CMOS resolution.

Structure	Temperature of the Chamber	View under the Microscope	Detail of the Error	Problem Description
Diamond	60 °C	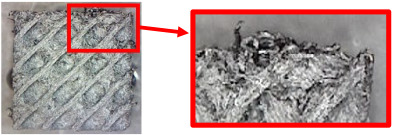		Tasseled surface around the edges of the sample
	65 °C	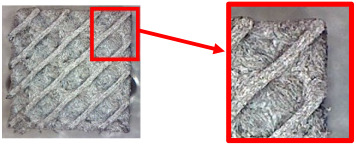		Better surface and layer continuity, and visibly improved sample integrity
	70 °C	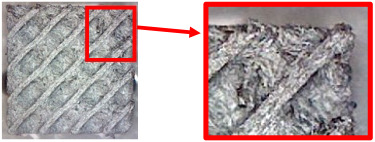		Visible deterioration of the sample surface compared to that under 65 °C
Gyroid	60 °C	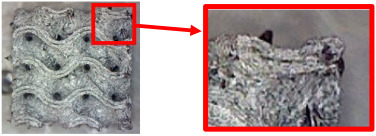		The surface shows a number of irregularities and dimensional changes after remeasurement
	65 °C	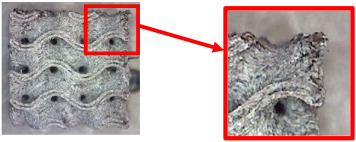		In contrast to that of the sample printed at 60 °C, the surface was more homogeneous
	70 °C	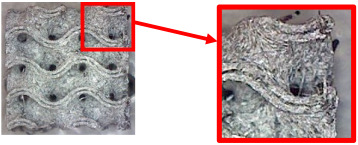		Larger gaps between layers in contrast to the print at 65 °C
Primitive	60 °C	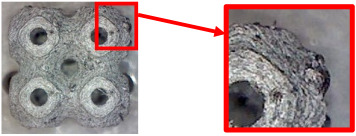		The surface contained a number of gaps and defects, which made the sample appear less whole
	65 °C	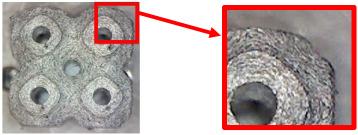		There were minimal defects on the samples, and the details were sharper
	70 °C	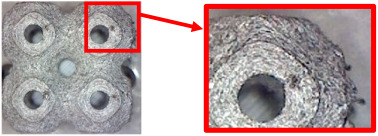		Compared with those under 65 °C, the edges were less compact (more fringed)

**Table 6:** Input parameters before printing started.

Input Parameter	Unit	Value
Filament diameter	mm	1.75
Temperature in the printer chamber	°C	65
Pad temperature	°C	65
Nozzle diameter	mm	0.4
Travel speed	mm/s	80
Print speed	mm/s	35
Layer height	mm	0.15

**Table 7:** Flexural modulus of elasticity.

Type of Structure	Modulus of Elasticity (MPa)	Bending Stiffness (N/m)	Flexural Rigidity (Nm^2^)
Schwarz Diamond	885	0.020087	2.808642
Schoen Gyroid	492	0.010011	1.669664
Schwarz Primitive	675	0.015454	2.378867

## Data Availability

Data are contained within the article.
